# Effective pretreatment of lignin-rich coconut wastes using a low-cost ionic liquid

**DOI:** 10.1038/s41598-022-09629-4

**Published:** 2022-04-12

**Authors:** Samson O. Anuchi, Kyra L. Sedransk Campbell, Jason P. Hallett

**Affiliations:** 1 Laboratory of Sustainable Chemical Technology, Department of Chemical Engineering, Imperial College London, South Kensington Campus, London, SW7 2AZ UK; 2 Department of Chemical and Biological Engineering, University of Sheffield, Sheffield, S1 3JD UK

**Keywords:** Chemical engineering, Crop waste

## Abstract

Coconut husks and shells are underutilised agricultural feedstocks in the bio-based industry. These biomass wastes have a higher lignin content than other woody biomass and have excellent potential as raw materials for the production of lignin-based materials. This work demonstrates the performance of a low-cost protic ionic liquid, *N,N,N*-dimethylbutylammonium hydrogen sulfate ([DMBA][HSO_4_]), for ionoSolv pretreatment of coconut husk and shell at 150 °C for 45–90 min and 170 °C for 15–60 min. Optimum pretreatment conditions were observed at 170 °C and 45 min for both feedstocks. At these conditions, [DMBA][HSO_4_] was able to remove almost 77 wt% of the lignin from the husk; leaving a cellulosic rich pulp behind, which released 82 % of the theoretical maximum glucose after enzymatic saccharification. The pretreated shell, by comparison, achieved 82 wt% lignin removal and 89 % glucose yield and these higher values could be attributed to the highly porous structure of coconut shell cell walls. The cleavage of the β-O-4 aryl ether linkages of lignin followed by extensive C–C condensation in the lignin at longer pretreatment times was shown by HSQC NMR analysis. This extensive condensation was evidenced by molecular weights > 10,000 g/mol exhibited by lignin precipitated after pretreatment at high temperature and long times. The high degree of lignin removal and high glucose release from both feedstocks demonstrate that [DMBA][HSO_4_] is an excellent ionic liquid for fractionation of very lignin-rich biomass.

## Introduction

Production of biomass-derived petroleum alternatives has attracted significant academic and industrial interest^1^. There is a need in the bio-based industry to expand lignocellulosic feedstocks beyond forest woods and herbaceous plants towards agricultural residues^2^. Agricultural residues are amongst the most abundant feedstocks globally^3^. These residues include straws, husks, cobs, and stalks, which are all generated as wastes during industrial agricultural processing^4^; other underutilised agricultural biomass, namely horticultural endocarp drupes (fruits) residues^5^. These may include the hardened inedible parts of the fruit that encloses the seed (endocarp) or the fleshy part of the fruit (mesocarp) that lies in between the endocarp and outer layer (exocarp)^6^. Endocarp drupe residues are a very rich source of lignin (up to 50 wt%); therefore, they are potentially a high-energy feedstock for the production of fuels and value-added chemicals and materials^5,7^. Globally, about 24–31 million tonnes of endocarp drupe residues are produced per annum, which majorly driven by coconut production^6,8^.

Coconut is an agriculturally produced endocarp drupe from the coconut palm (*Cocos nucifera*). As an economically important plant in the palm family, the coconut palm is cultivated as a food crop or for ornamental purpose^9^. Coconut palm is grown in more than 92 countries on more than 11 million hectares of land with the global production of coconut estimated at 62.5 million tonnes^9–11^. The largest coconut producers are Indonesia, the Philippines, India, Brazil, Sri Lanka, and Vietnam^10^. Some of these countries rely on coconuts for exportation, thus playing important economic and cultural roles^9^.

Coconut is a monospermous (one-seed) fruit which contains three major layers: a green, yellow, orange, brown or reddish-brown exocarp, a fibrous mesocarp (husk), and a very hard endocarp (shell). The shell encloses the white layer seed or meat (copra), which contains liquid coconut water^9^. Mature coconuts have an average weight of 1.2–2.5 kg or more and an average length of 20–30 cm, where the husk accounts for 40 wt% and the shell 30 wt%^12^. These parts are generated as wastes from the industrial processing of coconut for commercial production of its copra, water or oil^13^. Currently, coconut wastes are burnt as fuel to generate low-grade heat for cooking in many low-income countries. Limited small-scale product generation from the waste has also been reported, including for use in mats, ropes, and binder-less boards^13,14^. More developed applications have been explored to exploit coconut wastes. In particular, high-quality activated carbons^15^, fillers for natural rubber and concrete^16^ are derived from coconut shells.

Presently, the world is focusing on the sustainable conversion of lignocellulosic biomass wastes to fuel, chemicals, and materials^10,13^. Chemically, coconut husks and shells are similar to other lignocellulosic biomass, which are primarily composed of cellulose, hemicellulose, and lignin. On a dry weight basis, coconut wastes contain 20–30 wt% cellulose, 15–30 wt% hemicellulose, and nearly 50 wt% lignin^17–19^. Whilst other biomass feedstocks, with high cellulose and hemicellulose (and low lignin) are desirable for paper and biofuel production^20^, coconut, with increased lignin content is ideally suited for the production of lignin-based materials such as carbon fibres^21^ and activated carbons^22^.

Before biochemical conversion (towards fuels, materials, and chemicals) can take place, pretreatment is required to break down (fractionate) lignocellulosic feedstocks into separate streams of carbohydrates (derived from cellulose and hemicellulose) and lignin^20^. A breadth of pretreatment methods have been studied on agricultural feedstocks, including the use of alkalis, dilute acids, organic solvents, and ionic liquids^23^. Whilst alkaline and acid pretreatments are the most common, these methods are not effective at fractionating very rich lignin feedstocks (such as coconut shells and husks)^24^. Organic solvent use, i.e., organosolv pretreatment, is expensive and highly flammable due to the inherent risks associated with the solvents and therefore reduces its viability from a safety standpoint^25^. Ionic liquid pretreatment is one of the most promising alternative technologies for its simultaneous economic viability and efficacy^26^.

Ionic liquids (ILs) are salts that are liquids at < 100 °C^20^. Unlike conventional organic solvents, ILs have very low vapour pressure, non-flammability and high thermal stability^27^. These properties make ILs environmentally safe to handle as well as being reusable and recyclable^28^. Biomass pretreatment using ILs was initially focused on cellulose-dissolving (aprotic) solvents and amongst these is 1-ethyl-3-methylimidazolium acetate, [Emim][OAc], which has been the most commonly used IL for this process^4,29^. However, this imidazolium-based IL is very expensive; thermally unstable and most effective at very low water (< 1 wt%) content of the IL-biomass mixture^28^. Deep eutectic solvents (DESs), which share some similar properties to ILs are less expensive and more benign than some ILs^5,30^. These solvents, DESs are usually formed by H-bond interactions between H-bond donor and H-bond acceptors^30–32^. Studies have reported the use of DESs for biomass^30–33^. However, DESs are thermally unstable at high temperatures, which limit their ability to deconstruct very-rich lignin feedstocks^31,34,35^.

The IL pretreatment technology, ionoSolv^36^, dissolves lignin and hemicellulose in an aqueous protic IL solution, leaving a cellulose-rich pulp behind. Subsequently, an anti-solvent (e.g., water) is added to precipitate the lignin out of the IL liquor^20^. IonoSolv pretreatment uses cheap protic (acidic) ILs, which are easily synthesized by mixing an acid with a base in one-step reaction^37^.

A family of alkylammonium hydrogen sulfate ILs are amongst the class of protic ILs that was developed by Hallett et al.^38^. These protic ILs are more thermally stable than [Emim][OAc] and can be recycled by distillation and reused^4,39^ and are most effective at 20–40 wt% of water content to increase biomass delignification and saccharification yield^40,41^. One common member is triethylammonium hydrogen sulfate ([TEA][HSO_4_]), whose (2020) production cost can be as low as $0.78 kg^−1^ ^42^, > 80 times cheaper than the aprotic [Emim][OAc]^38,39^. Previous studies have shown that [TEA][HSO4] can effectively delignify *Miscanthus*^28^, willow^41^, and sugarcane bagasse^4^, resulting in saccharification yields of over 80 % from air-dried pulps. Another low-cost protic ILs is *N*,*N,N*-dimethylbutylammonium hydrogen sulfate ([DMBA][HSO_4_]), which has low viscosity and been shown to dissolve and remove more lignin from softwood pine than [TEA][HSO_4_]^43^. Furthermore, [DMBA][HSO_4_] showed robust performance when used to deconstruct post-consumer pinewood up to six pretreatment cycles^44^. Therefore, [DMBA][HSO_4_] can be a solvent of choice to dissolve and separate the enormous quantities of lignin from cellulose-rich pulps in the cases of coconut husk and shell.

Limited studies have been carried out on ionic liquid pretreatment of coconut husks^45,46^ and coconut shells^47^. Sangjan and Widjaja^45^ pretreated coconut husks using 1,3-dimethylimidazolium dimethyl phosphate (aprotic IL) at 120 °C for 15 h and achieved only < 20 wt% removal of cellulose with no delignification. Zakaria et al.^47^ extracted only < 10 wt. % of lignin from coconut shell after pretreatment with [Emim][OAc] at 150 °C for 2 h.

This study investigated the performance of a low-cost IL [DMBA][HSO_4_] for the fractionation of these lignin-rich biomass feedstocks. IonoSolv pretreatment was employed at 150 and 170 °C for different durations to determine the optimum pretreatment conditions. Delignification and enzymatic glucose release were both used as key performance indicators to assess pretreatment performance. Composition of the pretreated biomass pulps and the structural properties and molecular weight distribution of the isolated lignins were determined. After pretreatment, the properties of lignin are altered. The ionoSolv process can have a huge impact on lignin properties and this impact depends on factors such as temperature, pretreatment time and acid:base ratio (a/b) of the IL^48,49^.

## Materials and methods

Coconut husks and shells were collected from coconuts purchased from the ZAROS Fresh Fruit Market at Brixton, London, United Kingdom. These coconuts were mature Tall variety of brown fruits that were exported from Costa Rica. Sample preparation of the feedstocks was carried out following the National Renewable Energy Laboratory (NREL) procedure on the preparation of samples for composition^50^. After collection, the feedstocks were air-dried at room temperature for 48 h. Each feedstock was ground using a cutting mill (Retsch SM200, Germany) and then sieved using a vibratory shaker (Retsch AS200, Germany) to a 0.18–0.85 mm particle size. The ground shells and husks were then stored in sealed plastic bags. Mass measurements were taken using a VWR analytical balance LA 214i (± 0.0001 g). All chemical reagents used in this study were purchased from VWR International and Sigma-Aldrich and used as received unless otherwise noted.

### Ionic liquid synthesis

All pretreatment experiments were conducted in a *N*,*N,N*-dimethyl butylammonium hydrogen sulfate {[DMBA][HSO_4_]} with an acid: a base ratio of 1:1 (mol/mol) was synthesized according to the method published by Gschwend et al.^43^. The water content of the synthesized [DMBA][HSO_4_] was adjusted to 20 wt% using a V20 Volumetric Karl-Fischer titrator (Mettler-Toledo, USA).

### IonoSolv pretreatment and lignin recovery

Pretreatment of the husks and shells were carried out in triplicate according to the standard operating procedure of our laboratory^36^. The process involved heating 10 g/g of a [DMBA][HSO_4_]: feedstock mixture with 20 wt% water content in an ACE pressure tube at 150 and 170 °C for 15–90 min. After each pretreatment, the cellulose-rich pulps were separated and washed four times using 40 mL of ethanol. The pulps were then Soxhlet extracted for 24 h using ethanol. The collected Soxhlet ethanol was combined with the previous ethanol wash and evaporated to form a viscous IL liquor. Lignin was precipitated out of the IL liquor after several additions of water (anti-solvent). The isolated lignins were dried using a freeze-dryer for 48 h.

### Compositional analysis

Determination of structural carbohydrates, lignin and ash of the husks, shells and the pretreated pulps was carried out according to the NREL published protocols^51^. The detailed procedure can be found in the “Electronic Supplementary Information (ESI)”.

### Saccharification assay

Saccharification assays were carried out on untreated biomass and air-dried pulps in triplicate according to the NREL published protocol (Low Solids Enzymatic Saccharification of Lignocellulosic Biomass)^52^. A 100 ± 5 mg of air-dried biomass was weighed and placed in a Sterilin tube. To correct for sugar residues in the enzyme solution, three blanks were run with 100 µL of purified water. A 9.9 mL enzyme solution was used for each sample. This enzyme solution was prepared by mixing of 5 mL of 1 M sodium citrate buffer at pH 4.8; 30 μL of 10 mg/mL cycloheximide antibiotic: water solution; 40 μL of 10 mg/mL tetracycline antibiotic: 70 % ethanol solution; 4.78 mL purified water and 50 μL of CellicR CTec2 enzymes (Novozymes, Denmark). All saccharification samples were incubated in a Stuart Orbital Incubator (S1500) at 50 °C and 250 rpm for 7 days. Thereafter, all saccharification mixtures were filtered through 0.2 µm through a PTFE syringe filter and their filtrates were run on a Shimadzu HPLC with an AMINEX HPX-97P column (Bio-rad, 300 × 7.8 mm) with purified water as mobile phase (0.6 mL/min). The column temperature was 85 °C and acquisition was run for 20 min. Calibration standards with concentrations of 0.1, 1, 2 and 4 mg/mL of glucose, xylose, mannose, arabinose and galactose and 8 mg/mL of glucose were used. Glucose yields were calculated relative to the total glucan content of untreated biomass.

### Elemental analysis

Carbon, hydrogen, nitrogen and sulfur (CHNS) elemental analyses were carried out in triplicates using a Vario MICRO element analyser. Both untreated feedstocks were analysed on an air-dried and ash-free basis. The oxygen content for each run was estimated by subtracting the sum of C, H, N, and S (wt%) from 100%.

### Lignin characterization

Heteronuclear Quantum Coherence (HSQC) NMR spectroscopy of isolated lignin carried out in DMSO-d^6^, and NMR spectra were recorded on a Bruker 600 MHz spectrometer (pulse sequence hsqcetgpsi2, the spectral width of 10 ppm in F2_2_(^1^H) with 2048 data points and 160 ppm in F1_1_ (^13^C) with 256 data points, 16 scans and 1 s interscan delay).

Fourier-transform infrared spectroscopy (FT-IR) was used to investigate the lignin functional groups. The FT-IR spectra in the region of 4000–500/cm was collected on a Cary 630 FTIR spectrometer equipped with an attenuated total reflectance (ATR) cell at 2/cm resolution.

Gel permeation chromatography (GPC) was used to determine the molecular weight distributions for the isolated lignin, from both the pre-treated husk and shell. These measurements were performed in duplicates using an Agilent 1260 Infinity instrument equipped with a Viscotek column set (AGuard, A6000 M and A3000 M). An Agilent 1260 Infinity RID detector was used for detection. GPC grade DMSO containing LiBr (1 g/L) was used as eluent at a flow rate of 0.4 mL/min at 60 °C. Samples were prepared by dissolving 15 mg lignin in 1.5 mL eluent and filtering through a 0.2 μm syringe filter. Ten pullulan standards (Agilent calibration kit, 180 < M_p_ < 780 000) were used to calibrate the instrument.

## Results and discussion

A comparison of the composition and elemental properties of both shell and husk before pretreatment indicates differences (Table 1), notably in the carbohydrate profiles. The husk contained *ca*. 38 wt% cellulose, as compared to only *ca*. 25 wt% in the shell. Coconut husks are fibrous biomass, resembling sugarcane bagasse and rice straw, which also contain higher cellulose content than endocarp biomasses (e.g., coconut shells, palm kernels, and pistachio nuts)^5^. By contrast, the hemicellulose content is higher in the shell, 28 wt%, than the husk, 15 wt%. Also, the lignin content is slightly higher in the shell, 46 wt%, than the husk, 41 wt% and these results are consistent with the literature^16,18,53–56^. By contrast to herbaceous biomass, total carbohydrate content in both feedstocks (52 %) are much lower than herbaceous biomass (> 69 %)^4^, pointing toward lignin valorisation as a key application. Elemental (CHNS and O) analysis revealed that the husk, which has higher ash content, contained comparable carbon content to that of the shell (Table 1).

**Table 1:** Elemental and Chemical composition of coconut residues (based on dry wt. % basis) investigated in this study (Error bars represent standard deviations for three replicate measurements).

	Coconut husk (wt%)	Coconut shell (wt%)
**Compositional analysis**		
Cellulose	37.6 ± 1.1	25.2 ± 1.0
Hemicellulose	15.2 ± 0.9	27.7 ± 0.3
Lignin	41.3 ± 1.6	46.0 ± 0.8
Extractives	3.4 ± 0.1	1.0 ± 0.0
Ash	2.5 ± 0.1	0.1 ± 0.0
**Elemental composition**		
C	49.9 ± 0.1	52.0 ± 0.1
H	5.4 ± 0.0	5.5 ± 0.0
N	0.4 ± 0.1	0.3 ± 0.1
S	–	–
O^a^	44.3 ± 0.1	42.2 ± 0.1

^a^Calculated by difference (O = 100–C–H–N–S, wt%).

### Pretreatment and saccharification effectiveness for husk and shell

**Figure 1:**
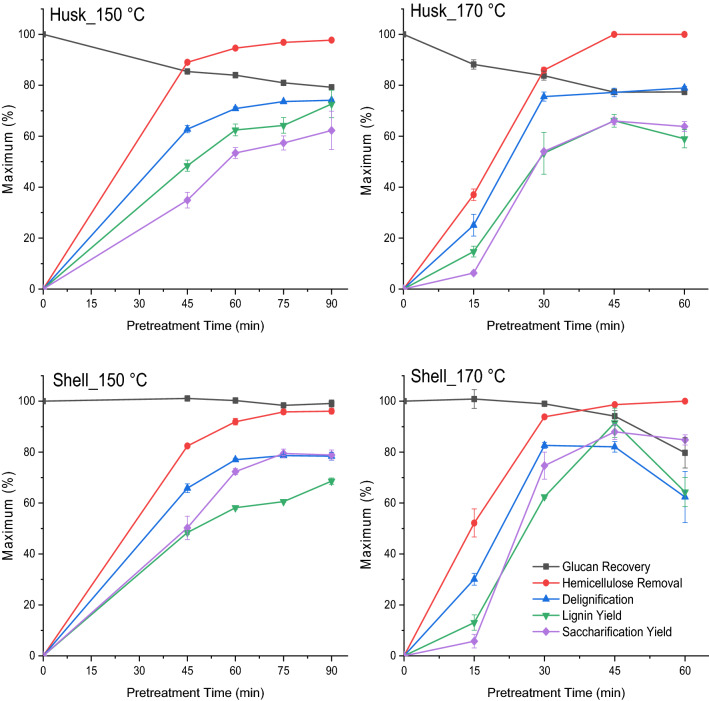
Performance indicators for ionoSolv pretreatment of husk and shell with [DMBA][HSO_4_] (20 % water content) in 1:10 g/g biomass: solvent loading at 150 for 45–90 min and 170 °C for 15–60 min (Error bars represent standard deviations for three replicate measurements).

To fractionate these lignin-rich feedstocks, the husks and shells were both treated with [DMBA][HSO_4_] ILs at either 150 or 170 °C^4^. Exposure duration was 45–90 min at 150 °C and 15–60 min at 170 °C to establish the optimal pretreatment conditions (Fig. 1).

Compositional analysis of the pulps (Fig. 1) reveals a rapid removal of lignin (25–85 wt% delignification) and hemicellulose (40–100 wt%) from the husk and shell by the protic ILs at both temperatures over time. This removal led to > 10% reduction of glucan content remaining in the pulps after pretreating both feedstocks for longer times. A continuous increase in lignin yield (45–70 wt%) was observed after pretreatment of husk and shell at 150 °C from 45 to 90 min. At 170 °C, lignin yields decreased gradually for husk (66–60 wt%) and rapidly for the shell (92–64 wt%) after 45 min. We attribute this lignin yield reduction at higher temperatures and longer times to lignin being re-deposited onto the cellulosic-rich pulp. The deposited lignin reduces the enzymatic hydrolysis of pulp to glucose and this effect has been reported in our previous work^4,41,49^. Here we extracted 73 wt% of the theoretical maximum amount of lignin from husk at 150 °C for 90 min and 92 wt% of the lignin from the shell at 170 °C for 45 min. Lignin yield for shell at 170 °C for 45 min slightly exceeded the delignification. This slight difference may be ascribed to the condensation of dissolve lignin and pseudo-lignin formation. Previous reports have shown that carbohydrate degradation products (e.g., 5-HMF and furfural), can react with lignin molecules forming polymers that resemble lignin (pseudo-lignins)^43,57,58^. Pseudo-lignin can be detected as acid-insoluble lignin in the composition analysis of the pretreated pulps^57,58^.

**Figure 2:**
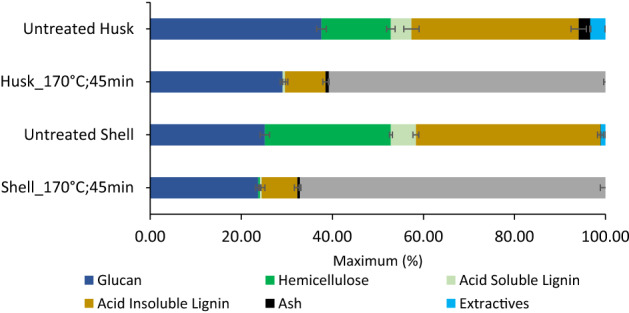
Chemical composition of untreated and pretreated shell and husk pulps before and after pretreatment with [DMBA][HSO_4_] ILs at optimum temperature and time conditions (Error bars represent standard deviations for three replicate measurements).

At both temperatures, a correlation exists between lignin removal (delignification) and glucose release (saccharification) from both feedstocks during pretreatment (Fig. 1). The maximum delignification and glucose release were achieved at 170 °C for 45 min for both feedstocks. The shell was initially suspected to be more recalcitrant towards fractionation than the husk due to its higher lignin content (Table 1). However, lignin removal for the shell (82 wt%) was more than that for the husk (77 wt%). Also, the shell resulted in higher glucose yield (88 wt%) than the husk (66 wt%) after enzymatic saccharification. We attributed this to the highly porous structure and brittleness of the cell wall structure of coconut shell, which is commonly found amongst endocarp tissues^4,5^. It can be said that fractionation is much easier and less energy-intensive than the husk. The changes in the composition to both the husk and shell as a result of pretreatment with [DMBA][HSO_4_] are optimized at 170 °C after 45 min (Fig. 2). Lignin removal from the shell and husk is comparable to that from rice husk using [TEA][HSO_4_] and much higher than those reported from IL-pretreatment of coconut biomass (Table S2)^4,45,47,59^.

Figure 2 shows that pretreatment using [DMBA][HSO_4_] at 170 °C for 45 min, removed more than 99 wt% of hemicellulose from both the husk and shell samples; glucan content in the untreated shells was comparable to the amount in shell pulps after pretreatment, whereas glucan content in the untreated husks decreased by 20% after pretreatment. At the optimum condition, [DMBA][HSO_4_] was able to produce cellulose-rich pulps that contained only 8 and 9 wt% of lignin remaining in shells and husks after pretreatment. This highlights the high quality of the pulps for enzymatic digestibility. Whilst a small ash fraction for the untreated material was recorded, > 70 % ash extraction from the husks was achieved (Table S1). This is likely due to the acid-solubility of the ash which then dissolves into the ILs^4^. By contrast, an increase in ash content (0.1–0.5 %) was observed after pretreatment of the shell, possibly due to precipitation of inorganic sulfate salts (e.g., CaSO_4_) onto the pulps^28^. The quantity of mineral ash found in the shell (0.5 %) did not impact the digestibility of the cellulose-rich pulps by the enzymes due to the amount of glucose released (89 wt%).

### Lignin characterisation

The previous section demonstrated the effects of delignification on enzymatic saccharification of cellulosic-rich pulps. Optimisation of glucose released by cost-effective delignification of biomass has been one of the major objectives of lignocellulosic biorefineries. However, optimum valorisation of lignin isolated from biomass may improve biorefinery economics. Consequently, lignins extracted from the husks and shells were analysed to understand the impacts of ionoSolv pretreatment on the sub-unit composition and structural properties and the molecular weight distribution of the lignin. This will help in defining future applications for the lignins.

### Lignin structural properties

**Figure 3:**
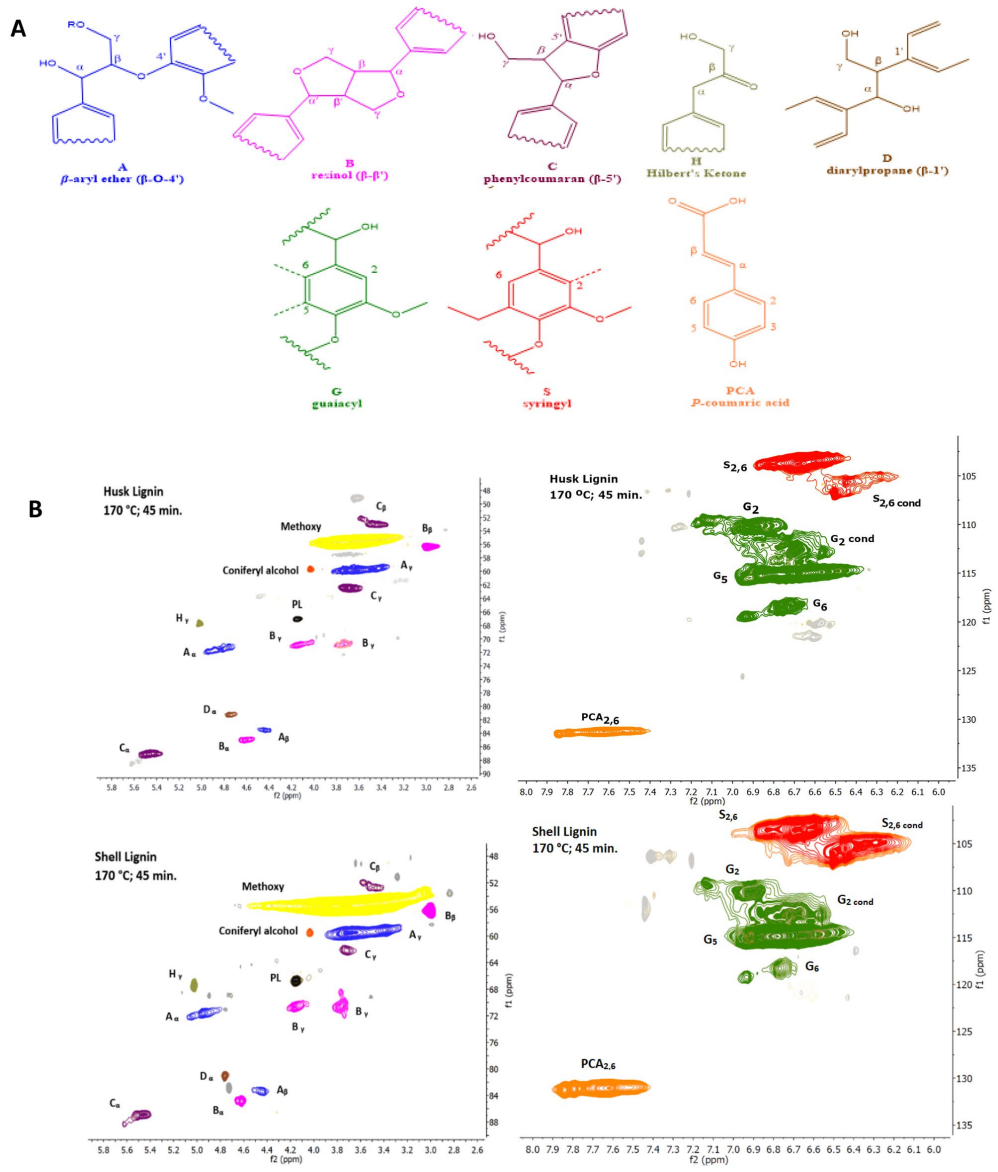
(**A**) Important inter-unit linkages and subunits found in the husk- and shell-lignin, and (**B**) HSQC NMR side-chain (left) and aromatic spectra regions (right) units for lignins isolated from 1:10 g/g biomass (husk and shell) and [DMBA][HSO_4_] with 20 % water at 170 °C for 45 min.

Lignins isolated at the optimised pretreatment conditions at (170 °C and 45 min) were analysed with HSQC NMR spectroscopy, to establish the lignin subunits and linkages present. Figure 3A shows the important structural subunits and linkages found in husk and shell lignin, while the spectra regions are presented in Fig. 3B and Fig. S1 for additional data.

**Figure 4:**
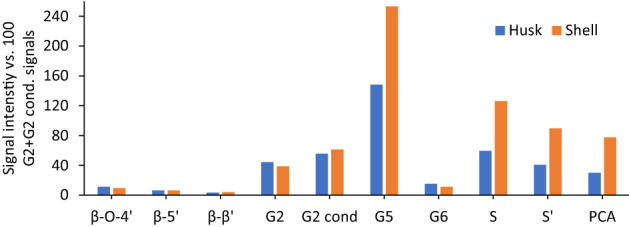
Abundance of HSQC NMR signal intensities of different lignin C–H units relative to 100 G2 + G_2,cond_ units for lignin isolated from 1:10 g/g biomass (husk and shell) and [DMBA] [HSO_4_] with 20 % water at 170 °C for 45 min durations (monoplicate measurement).

The amounts of husk- and shell-lignin subunits and linkages were estimated from the raw HSQC NMR spectra (Fig. 4) using a semiquantitative technique^28,43^. All signal intensities were normalized to 100% abundance of the sum of G_2_ and G_2,Cond_, which was suggested as the internal reference standard by Brandt-Talbot et al*.*^28^ for grasses and Gschwend et al.^43^ for softwoods.

The side chain regions of the NMR spectrum (Fig. 3B) revealed ca 11 % of β-O-4 ether linkages remained in husk lignin and ca 9 % in shell lignin (Fig. 4) isolated after pretreatment using [DMBA][HSO_4_] at 170 °C for 45 min. Both β-β (4.6/85.0 ppm) and β-5 (5.5/87.2 ppm) are observed in the NMR side-chain region (Fig. 3B), although the NMR signal intensities are very low for the husk lignin and shell lignin. These linkages are found in resinol (B: β-β + α-O-γ) and phenylcoumaran (C: β-5 + α-O-4) substructures of lignin^60^. About 3% of β–β linkages and 6% of β-5 linkages were found in the lignins isolated from the husk and shell (Fig. 4). The amount of β-5 linkages in the phenylcoumaran substructure for the lignins is almost half the value (13%) reported by Rencoret et al.^61^ for milled wood lignins isolated from coconut husk. It was suggested by Brandt et al.^48^ that β-5 could have been modified in the acidic media at that high temperature and long pretreatment times, rather than linkage cleavage, as these C–C linkages do not easily break.

A signal peak at 4.2/67 ppm (Fig. 3B) can be assigned to bis (2-ethylhexyl) phthalate (leached O-ring plasticiser)^43^. Additionally, a signal for Hibbert’s ketone (H_γ_) is detected at 67.0/4.2 ppm (Fig. 3B). This keto-containing structure is a reactive intermediate (product) formed during the hydrolysis of β-O-4 ether cleavage. This product easily re-polymerises and form stronger bonds thus, promoting lignin condensation^62^. This reactive product has been similarly observed in walnut and peach^5^ as well as *Miscanthus *× *giganteus*^48^.

The aromatic region of the spectrum (Fig. 3B) revealed pronounced intensities in G_2,Cond_ and S_Cond_ subunits of lignins isolated from the husk and shell, which supported C–C condensation of lignin macromolecules that occurs at the 2- and 6-aromatic ring positions of G and S lignin subunits^48^. This is evidenced by the number of G_2,Cond_ (56 and 61%) and S_Cond_ (49 and 90%) subunits the husk lignin and shell lignin respectively (Fig. 4). It was also observed that the number of S subunits was twice higher in the shell lignin (*ca* 120%) than the husk lignin (*ca* 60%) (Fig. 4). The strong predominance of S-lignin subunit in the shell lignin was evidenced by S/G ratio > 1.0 (Table 2). Based on the S/G ratio, shell lignin resembled hardwood due to the abundance of syringyl lignin subunits. About 150 and 250% of G_5_ subunits were determined in the husk lignin and shell lignin respectively (Fig. 4). This may be attributed to pseudo-lignins, which are usually formed and precipitated with lignin during pretreatment in acidic media at high temperature and prolonged time^43^.

**Table 2:** Degree of condensation evidenced by S/G ratios for lignins isolated from 1:10 g/g biomass (husk and shell) and [DMBA] [HSO_4_] with 20% water at 170 °C for 45 min.

	Husk lignin	Shell lignin
S2 (%)	59.3	126.3
S, Cond (%)	40.6	89.6
G2 (%)	44.3	38.7
G2, Cond (%)	55.7	61.3
S/G ratio^a^	0.7	1.5

^a^Syringyl to Guaiacyl ratio.

In addition to HSQC NMR analysis, FT-IR spectroscopy was used to examine the structural properties (functional groups) of the shell and husk lignin samples, which are presented in Fig. S2. The IR spectra of both lignin samples are almost similar, and their absorption assignments are based on the literature^63^. Both lignin samples exhibited a broad band at 3365/cm, which represent O–H stretching vibrations in aliphatic and phenolic O–H groups. While the peaks at 2935 and 2842/cm represent CH_n_ bonds for alkane side chains. Aromatic skeletal vibration was found at 1592, 1508, and 1458/cm and C–C, C–O, and C=O stretching were found at 1267 and 1213/cm. The bands at 1159 and 1113/cm were connected with guaiacyl (G) and syringyl (S) subunits of lignin, respectively^5,64^. The more intense band at 1113/cm for the shell lignin than husk lignin corresponds to the NMR analysis (Table 2). Moreover, the intense band at 1701/cm indicates the presence of α-carbonyl, unconjugated C=O units. This may be attributed to the presence of carbohydrate degradation products in the lignin samples^57,58^.

#### Lignin molecular weight distribution

Molecular weight, an important structural property alongside lignin composition (inter-unit linkages and subunits), affects the reactivity and potential valorisation of lignin after isolation from biomass pretreatment^20,65^. Lignin molecular weights were estimated from the GPC analysis by two markers: the number of average molecular weight (M_n_) and weight average molecular weight (M_W_).

**Figure 5:**
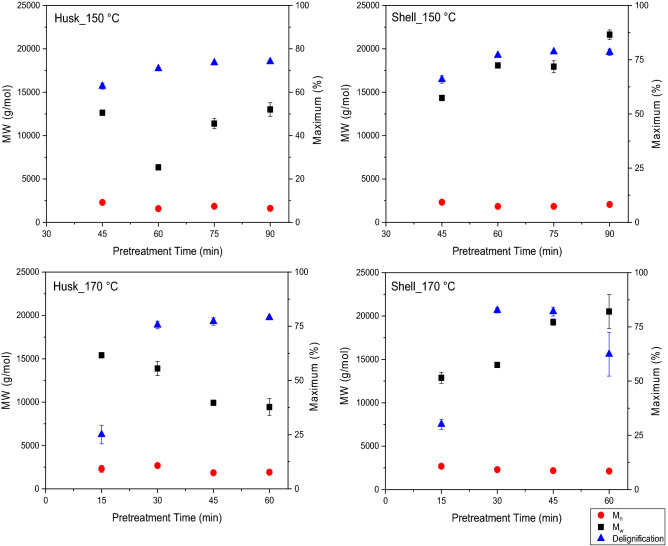
Relationship between biomass delignification and molecular weight distribution for lignins isolated from the IonoSolv pretreatment of 1:10 g/g biomass (husk and shell) and [DMBA][HSO_4_] with 20 % water at 150 °C for 30–90 min and 170 °C for 15–60 min (Error bars represent standard deviations for two replicate measurements).

Figures S3 and S4 (“Supplementary information”) present the chromatograms of lignin isolated after each pretreatment. Figure 5 summarizes the relationships between the molecular weight of isolated lignins and the biomass delignification at 150 for 45–90 min and 170 °C for 15–60 min.

At 150 °C, an initial decrease in M_n_ for lignins isolated from the husk (2299–1584 g/mol) and shell (2320–1836 g/mol) as well as a sharp decrease in M_w_ for husk lignin (13,000–6350 g/mol) were observed as pretreatment time increased to 60 min (Fig. 5). This indicates that the lignin macromolecules undergo depolymerisation through the cleavage of their aryl ether C–O linkages as also observed for willow by Weigand et al.^41^. After 60 min, no significant change in M_n_ was observed for the husk lignin and shell lignins, but M_w_ for husk lignin increased from 6350 to 13,000 g/mol as the pretreatment time approached 90 min. A steady increase in M_w_ occurred by the shell lignin (14,340–21,640 g/mol) as the pretreatment time increased to 90 min. It appears that the rate of re-polymerisation was occurring faster than depolymerisation at prolonged pretreatment times using [DMBA][HSO_4_] and resulted in the recovery of lignin with an M_w_ above 10,000 g/mol. This increase in M_w_ correlates with the biomass delignification. As the increase in the removal of lignin during pretreatment approached the threshold point at 75 min, the M_w_ of the isolated lignin increased further. We infer that pseudo-lignin (degraded carbohydrate-lignin complex) may have precipitated alongside the lignin, which increased lignin yields.

At 170 °C, the M_w_ of the lignin isolated from the husk rapidly decreased (15,400–9400 g/mol) as the pretreatment time approached 60 min (Fig. 5). We attribute this to depolymerisation of the lignin macromolecules by [DMBA][HSO_4_] at high temperatures. The M_w_ lignins isolated from the shell (12,800–20,500 g/mol) increased over the pretreatment time. This increase, which was also observed at 150 °C, maybe due to condensation of lignin macromolecules after prolonged pretreatment of the shell. Also, there was a further increase in shell lignin M_w_ after 45 min, even though delignification of the shell rapidly dropped. We infer that high pretreatment temperature, as well as the acidic ILs, may have contributed to re-condensation, re-precipiation and the higher molecular weight observed in lignin isolated from the shell.

## Conclusion

Coconut wastes, which include husk and shell, are underutilised feedstocks in the bio-based industry. These feedstocks have higher lignin content (> 40 wt%) than other biomass and are available in large quantities, suggesting them as a source for lignin-based materials. This study has demonstrated the performance of [DMBA][HSO_4_] ILs at fractionating coconut husk and shell to produce both carbohydrate and lignin streams. The ionoSolv process was employed to fractionate these coconut wastes using [DMBA][HSO_4_] at 150 and 170 °C for 15–90 min. The optimal pretreatment condition and was determined at 170 °C for 45 min based on the amount of glucose release via saccharification and delignification. At the optimum conditions, ionoSolv pretreatment was able to remove 77 wt% of lignin from the husk leaving a cellulosic-rich pulp that could release 66 wt% of glucose. The shell showed higher delignification (82 wt%) and saccharification (88 wt%) than the husk. Also, about 73 and 92 wt% of lignins were precipitated from the husk and shell respectively.

The pretreatment showed a great impact on the properties of lignin isolated from both feedstocks. A breakdown of *β*-O-4 ether linkages was observed in the HSQC NMR spectra with subsequent extensive condensation of lignin isolated at the optimum delignification. Shell lignin, which showed S/G ratio > 1 contained much more amount of syringyl lignin subunits than the husk lignin. We also observed a decrease in the M_n_ of lignins as the pretreatment times increased. Due to the extensive condensation, high molecular weight lignins were extracted from husk and shell.

## Supplementary Information

Supplementary Information.

## Data Availability

All data acquired for this study are presented in this published article and its supplementary information documents. Materials can be made available by request to the author.
