# Supplementary material for: Effective pretreatment of lignin-rich coconut wastes using a low-cost ionic liquid
Source: Sci Rep. 2022 Apr 12;12:6108. doi: 10.1038/s41598-022-09629-4 (PMC9005540; doi:10.1038/s41598-022-09629-4)
Supplement: Supplementary file 1 — Supplementary Information. [file 41598_2022_9629_MOESM1_ESM.docx]

**Electronic Supplementary Information**

**Effective Pretreatment of Lignin-rich Coconut Wastes Using a Low-Cost Ionic Liquid**

Samson O. Anuchi^1^, Kyra L. Sedransk Campbell^2^ and Jason P. Hallett^1🖂^

^1^Laboratory of Sustainable Chemical Technology, Department of Chemical Engineering, Imperial College London, South Kensington Campus, London, United Kingdom. ^2^Department of Chemical and Biological Engineering, University of Sheffield, Sheffield, S1 3., United Kingdom. ^🖂^email: j.hallett@imperial.ac.uk

***Compositional Analysis***

About 300 mg (on ODW basis) of air-dry biomass or pulp was weighed out into a 100 mL pressure tube and the weight recorded. 3 mL of 72% sulfuric acid was added, the samples stirred with a Teflon stir rod and the pressure tubes placed into a preheated water bath at 30 °C. The samples were stirred again every 15 min for one hour, they were then diluted with 84 mL distilled water sealed. The samples were autoclaved (Sanyo Labo Autoclave ML5 3020 U) for one hour at 121°C and left to cool. The samples were then filtered through filtering ceramic crucibles of a known weight. The filtrate was stored in two plastic tubes and the remaining residue washed with distilled water. The crucibles were placed into a convection oven (VWR Venti-Line 115) at 105°C for 24±2 hours. They were placed in a desiccator for 15 min and the weight recorded. The crucibles were then placed into a muffle oven (Nabertherm + controller P 330) and ashed to constant weight at 575°C. The crucible weight after ashing was recorded. The content of acid insoluble lignin (AIL) was determined according to equation 1.

$$\%AIL= \frac{Weightcrucible plus AIR ‒ Weightcrucible plus ash}{ODWsample}. 100 (eq.1)$$

where Weightcrucibles plus AIR is the weight of the oven-dried crucibles plus the acid insoluble residue, Weightcrucibles plus ash is the weight of the crucibles after ashing to constant temperature at 575°C.

The supernatant was used for the determination of acid soluble lignin content (ASL) by UV analysis at 240 nm (equation 2) using a Perkin Elmer Lambda 650 UV/Vis spectrometer.

$$\%ASL = \frac{A}{l \cdot\varepsilon\cdot c}.100= \frac{A \cdot Vfiltrate}{l \cdot\varepsilon\cdot ODWsample}.100 (eq. 2)$$

A is the absorbance at 240 nm, l is the path length of the cuvette in cm (1 cm in this case), ε is the extinction coefficient (12 L/g cm), c is the concentration in mg/mL, *ODW* is the oven-dried weight of the sample in mg and *Vfiltrate* is the volume of the filtrate in mL and equal to 86.73 mL.

Calcium carbonate was added to the second liquid fraction until pH 5 was reached. The liquid was passed through a 0.2 μm PTFE syringe filter and subsequently submitted to HPLC analysis (Shimadzu, Aminex HPX-97P from Bio-Rad, 300 x 7.8 mm, purified water as mobile phase at 0.6 ml/min, column temperature 85°C, de-ashing columns were used as pre-filters) for the determination of total sugar content. Calibration standards with concentrations of 0.1, 1, 2 and 4 mg/mL of glucose, xylose, mannose, arabinose and galactose were used. Sugar recovery standards were prepared as 10 mL aqueous solutions close to the expected sugar concentration of the samples and transferred to pressure tubes. 278 μL 72% sulfuric acid was added, the pressure tube closed and autoclaved and 3 the sugar content determined as described above. The sugar recovery coefficient (SRC) was determined according to equation 3 and the sugar content of the analysed sample using equation 4:

$$SRC = \frac{cHPLC \cdot V}{initial weight} (eq. 3)$$

$$\%Sugar = \frac{cHPLC \cdot V \cdot corranhydro SRC}{ODWsample}.100 (eqn. 4)$$

where *cHPLC* is the sugar concentration detected by HPLC, *V* is the initial volume of the solution in mL (10.00 mL for the sugar recovery standards and 86.73 mL for the samples), initial weight is the mass of the sugars weighed in, *corranhydro* is the correction for the mass increase during hydrolysis of polymeric sugars (0.90 for the C_6_ sugars glucose, galactose and mannose and 0.88 for the C5 sugars xylose and arabinose) and *ODW* is the oven-dried weight of the sample in mg.

***HSQC NMR Spectroscopic Calculations***

HSQC NMR Spectra collected were analysed using MestReNova (Version 8.0.0, Mestrelab Research 2012). All spectra were referenced to the DMSO peak at 2.500 ppm (^1^H) and 39.520 ppm (^13^C). Integrals were obtained for spectra of the same series of experiments simultaneously to ensure that the same areas were integrated. All relevant spectra were copied into one file and selected while integrating the peak areas in one spectrum. Integration areas were selected visually according to peak assignments found in the literature^28^ for ether linkages, the C–H_α_-signals were integrated. Integral sizes are reported relative to 100 (G_2_ + G_2, Cond_) signals. Syringyl/Guaiacyl ratio was calculated from HSQC NMR spectra signals using equation 5:

$$\boldsymbol{S/G}= \frac{\left( {0.5*S}_{2,6}+ S_{2,cond} \right)}{\left( G_{2}+ G_{2,cond} \right)} (eqn.5)$$

where *S_2,6_ and* *S_2,cond_* represent syringyl and condensed syringyl subunits; *G_2_ and G_2,cond_* represent guaiacyl and condensed guaiacyl subunits.

| Temp. (°C) | Time (Minute) | Glucan (wt.%) | Hemicellulose ^a^ (wt.%) | Acid Soluble Lignin (wt.%) | Acid Insoluble Lignin (wt.%) | Ash (wt.%) | Extractives (wt.%) | Mass loss ^b^ (wt.%) | Lignin ppt. ^c^ (wt.%) |
| --- | --- | --- | --- | --- | --- | --- | --- | --- | --- |
| Untreated Husk | | 37.6 ± 1.1 | 15.2 ± 0.9 | 4.5 ± 0.1 | 36.7 ± 1.7 | 2.5 ± 0.1 | 3.3 ± 0.1 | 0 | N/A |
| 150 | 45 | 32.1 ± 0.4 | 1.7 ± 0.1 | 1.0 ± 0.0 | 14.4 ± 0.3 | 0.4 ± 0.1 | 0 | 50.4 ± 0.7 | 17.7 ± 0.7 |
| 150 | 60 | 31.6 ± 0.3 | 0.8 ± 0.1 | 0.7 ± 0.0 | 11.3 ± 0.1 | 0.5 ± 0.0 | 0 | 55.1 ± 0.4 | 22.8 ± 0.7 |
| 150 | 75 | 30.5 ± 0.1 | 0.5 ± 0.0 | 0.6 ± 0.0 | 10.3 ± 0.1 | 0.5 ± 0.0 | 0 | 57.6 ± 0.1 | 23.5 ± 0.9 |
| 150 | 90 | 29.8 ± 0.2 | 0.3 ± 0.0 | 0.5 ± 0.0 | 10.2 ± 0.2 | 0.5 ± 0.0 | 0 | 58.7 ± 0.1 | 26.6 ± 1.6 |
| 170 | 15 | 33.2 ± 1.4 | 9.6 ± 0.2 | 2.8 ± 0.0 | 28.1 ± 1.0 | 0.8 ± 0.4 | 0 | 25.5 ± 1.8 | 5.4 ± 0.6 |
| 170 | 30 | 31.5 ± 0.6 | 2.1 ± 0.2 | 0.7 ± 0.0 | 9.9 ± 0.8 | 0.6 ± 0.1 | 0 | 55.6 ± 0.1 | 19.5 ± 2.5 |
| 170 | 45 | 29.1 ± 0.6 | 0 | 0.5 ± 0.0 | 8.9 ± 0.6 | 0.8 ± 0.0 | 0 | 60.7 ± 0.4 | 24.1 ± 0.7 |
| 170 | 60 | 29.1 ± 0.2 | 0 | 0.4 ± 0.0 | 8.3 ± 0.3 | 0.5 ± 0.2 | 0 | 61.7 ± 0.3 | 21.6 ± 1.1 |
| Untreated Shell | | 25.2 ± 1.0 | 27.7 ± 0.3 | 5.5 ± 0.2 | 40.5 ± 0.6 | 0.1 ± 0.0 | 1.1 ± 0.0 | 0 | N/A |
| 150 | 45 | 25.5 ± 0.5 | 4.9 ± 0.0 | 1.1 ± 0.1 | 11.0 ± 0.5 | 0.3 ± 0.0 | 0 | 57.2 ± 0.8 | 22.3 ± 0.3 |
| 150 | 60 | 25.3 ± 0.4 | 2.2 ± 0.3 | 0.6 ± 0.0 | 6.8 ± 0.2 | 0.1 ± 0.1 | 0 | 65.0 ± 0.5 | 26.8 ± 0.2 |
| 150 | 75 | 24.8 ± 0.2 | 1.2 ± 0.3 | 0.4 ± 0.0 | 6.4 ± 0.1 | 0.2 ± 0.0 | 0 | 67.0 ± 0.1 | 27.9 ± 0.1 |
| 150 | 90 | 25.0 ± 0.4 | 1.1 ± 0.3 | 0.4 ± 0.0 | 6.8 ± 0.5 | 0.3 ± 0.0 | 0 | 66.4 ± 0.5 | 31.6 ± 0.5 |
| 170 | 15 | 25.4 ± 0.9 | 13.2 ± 0.9 | 3.3 ± 0.1 | 28.9 ± 0.8 | 1.1 ± 0.5 | 0 | 28.1 ± 3.0 | 6.0 ± 1.1 |
| 170 | 30 | 24.9 ± 0.6 | 1.7 ± 0.2 | 0.6 ± 0.0 | 7.5 ± 0.4 | 0.6 ± 0.1 | 0 | 64.7 ± 0.7 | 28.8 ± 0.2 |
| 170 | 45 | 23.7 ± 0.1 | 0.4 ± 0.1 | 0.4 ± 0.0 | 7.9 ± 0.7 | 0.5 ± 0.2 | 0 | 67.1 ± 1.1 | 42.1 ± 2.2 |
| 170 | 60 | 20.4 ± 3.4 | 0.0 ± 0.0 | 0.3 ± 0.0 | 16.7 ± 3.4 | 0.7 ± 0.1 | 0 | 61.9 ± 3.3 | 29.6 ± 2.2 |

**Table S1.** Composition of husk and shell pulps as determined by compositional analysis, as well as lignin yield, after pretreatment with [DMBA][HSO_4_] with a biomass to solvent ratio of 1:10 g/g and a final water content of 20 wt.%.

**a** Sum of mannose, xylose, arabinose and galactose; **b** Mass dissolved in IL during pretreatment. **c** Lignin precipitate as percentage of initial biomass weight.

| Biomass | Ionic Liquid | Temp. (°C) | Time (Mins.) | Lignin Removal (%) | References |
| --- | --- | --- | --- | --- | --- |
| Coconut husk | [MMIM][DMP]^a^ | 120 | 900 | 0 | Sangjan and Widjaja^1^ |
| Coconut shell | [EMIM][OAc]^b^ | 150 | 120 | 10 | Zakaria *et al*.^2^ |
| Coconut husk | [EMIM][Cl]^c^ | 140 | 360 | 0 | Rambo *et al*.^3^ |
| Rice husk | [TEA][HSO_4_]^d^ | 170 | 45 | 80 | Chambon *et al*.^4^ |
| Coconut husk | [DMBA][HSO_4_] | 170 | 45 | 77 | This work |
| Coconut shell | [DMBA][HSO_4_] | 170 | 45 | 82 | This work |

**Table S2.** Comparison of lignin removal from ionoSolv pretreatment of the coconut husk and shell using [DMBA][HSO_4_] with rice rusk [TEA][HSO_4_] and other IL-pretreatment results in literature. ^a^[MMIM][DMP]: methyl methylimidazolium dimethyl phosphate. ^b^[EMIM][OAc]: 1-Ethyl-3-methylimidazolium acetate. ^c^[EMIM][Cl]: 1-Ethyl-3-methylimidazolium chloride. ^d^[TEA][HSO_4_]: triethylammonium hydrogen sulfate

***HSQC-NMR raw spectra***


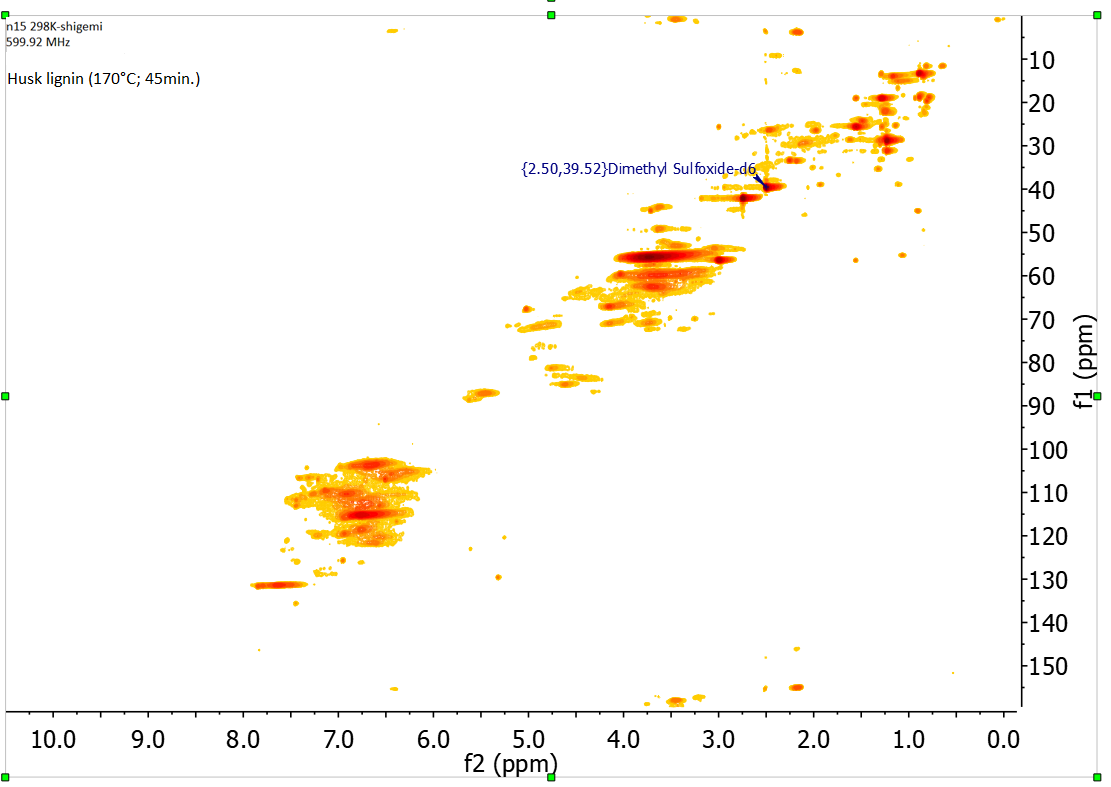


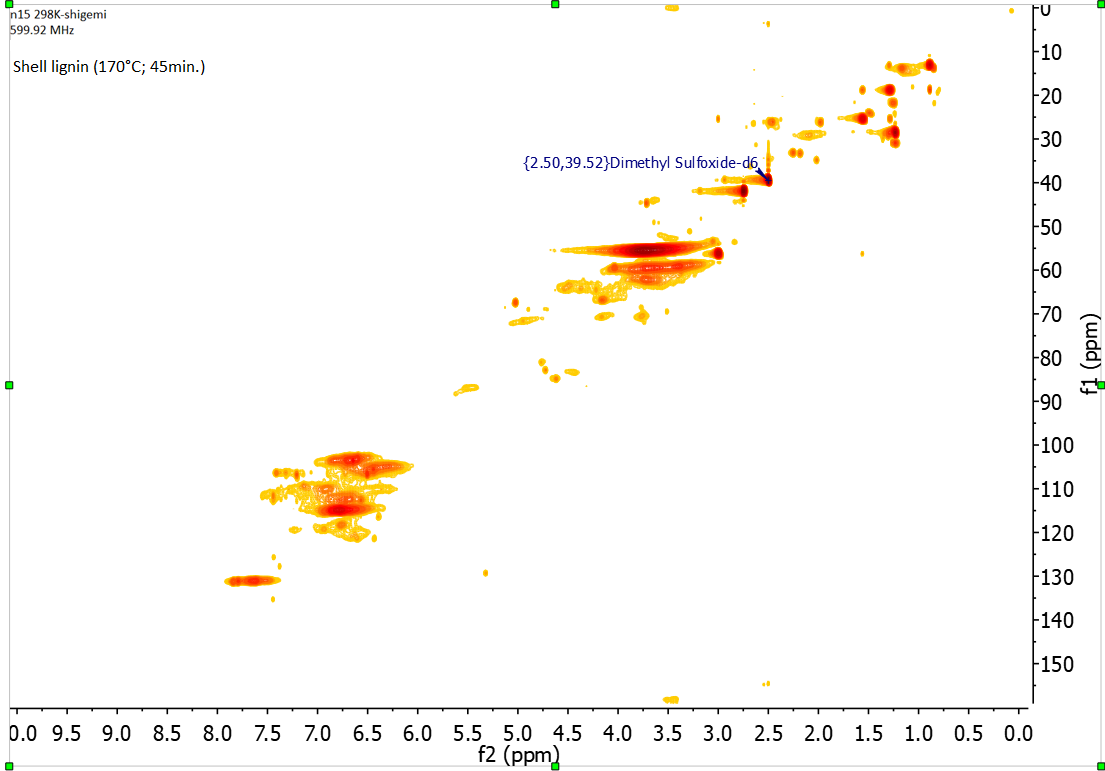


**Figure S1.** HSQC NMR full spectra regions for lignins isolated from 1:10 g/g biomass (husk and shell) and [DMBA][HSO_4_] with 20% water at 170 °C for 45 minutes.

***FT-IR spectra***

**Figure S2.** FT-IR spectra of lignins isolated from 1:10 g/g biomass (husk and shell) and [DMBA][HSO_4_] with 20% water at 170 °C for 45 minutes.

***Lignin Gel Permeation Chromatograms:***

**Figure S3.** Gel permeation chromatograms of lignins isolated from 1:10 g/g husk and [DMBA][HSO_4_] with 20% water at 170 °C after different pretreatment times.

**Figure S4**. Gel permeation chromatograms of lignins isolated from 1:10 g/g shell and [DMBA][HSO_4_] with 20% water at 170 °C after different pretreatment times.

**References**

1. Sangian, H. F. & Widjaja, A. Pretreatment of coir dust. *BioResources*. **12**, 8030–8046 (2017).

2. Zakaria, S. M., Idris, A. & Alias, Y. Lignin extraction from coconut shell using aprotic ionic liquids. *BioResources.* **12**, 5749–5774 (2017).

3. Rambo, M. K. D. *et al.* Optimization of Alkaline, Acidic, Ionic Liquid and Oxidative Pretreatments for Coconut Waste Conversion into Fermentable Sugars. *Journal of the Brazilian Chemical Society*. **31**, 904–915 (2020).

4. Chambon, C. L., Chen, M., Fennell, P. S. & Hallett, J. P. Efficient fractionation of lignin- and ash-rich agricultural residues following treatment with a low-cost protic ionic liquid. *Frontiers in Chemistry*. **7**, 1–13 (2019).
